# CytoSorb hemoperfusion markedly attenuates circulating cytokine concentrations during systemic inflammation in humans in vivo

**DOI:** 10.1186/s13054-023-04391-z

**Published:** 2023-03-21

**Authors:** Aron Jansen, Nicole J. B. Waalders, Dirk P. T. van Lier, Matthijs Kox, Peter Pickkers

**Affiliations:** 1 Department of Intensive Care Medicine, Radboud University Medical Center, Radboud Institute for Molecular Life Sciences (RIMLS), Nijmegen, The Netherlands; 2 Radboud University Medical Center, Radboud Center for Infectious Diseases (RCI), Nijmegen, the Netherlands

**Keywords:** Systemic inflammation, Hemoadsorption, CytoSorb, Extracorporeal therapy, Cytokines, Sepsis

## Abstract

**Background:**

The CytoSorb hemoadsorption device has been demonstrated to be capable of clearing inflammatory cytokines, but has not yet been shown to attenuate plasma cytokine concentrations. We investigated the effects of CytoSorb hemoperfusion on plasma levels of various cytokines using the repeated human experimental endotoxemia model, a highly standardized and reproducible human in vivo model of systemic inflammation and immunological tolerance induced by administration of bacterial lipopolysaccharide (LPS).

**Methods:**

Twenty-four healthy male volunteers (age 18–35) were intravenously challenged with LPS (a bolus of 1 ng/kg followed by continuous infusion of 0.5 ng/kg/hr for three hours) twice: on day 0 to quantify the initial cytokine response and on day 7 to quantify the degree of endotoxin tolerance. Subjects either received CytoSorb hemoperfusion during the first LPS challenge (CytoSorb group), or no intervention (control group). Plasma cytokine concentrations and clearance rates were determined serially. This study was registered at ClinicalTrials.gov (NCT04643639, date of registration November 24th 2020).

**Results:**

LPS administration led to a profound increase in plasma cytokine concentrations during both LPS challenge days. Compared to the control group, significantly lower plasma levels of tumor necrosis factor (TNF, − 58%, *p* < 0.0001), interleukin (IL)-6 ( − 71%, *p* = 0.003), IL-8 ( − 48%, *p* = 0.02) and IL-10 ( − 26%, *p* = 0.03) were observed in the CytoSorb group during the first LPS challenge. No differences in cytokine responses were observed during the second LPS challenge.

**Conclusions:**

CytoSorb hemoperfusion effectively attenuates circulating cytokine concentrations during systemic inflammation in humans in vivo, whereas it does not affect long-term immune function. Therefore, CytoSorb therapy may be of benefit in conditions characterized by excessive cytokine release.

**Supplementary Information:**

The online version contains supplementary material available at 10.1186/s13054-023-04391-z.

## Introduction

With mortality estimates of over eleven million deaths annually, sepsis represents one of the leading causes of death worldwide (1). Its pathophysiology is highly complex, with immunological derangements varying from profound hyperinflammation, characterized by excessive cytokine production, to a severe immunotolerant state, rendering the host unable to clear the primary pathogen and vulnerable toward secondary infections. This latter phenotype is also referred to as sepsis-induced immunoparalysis (2, 3). We and others have previously demonstrated that the initial release of pro- and anti-inflammatory mediators is instrumental for the induction of immunological tolerance, both in vitro (4) and in vivo (5, 6). As such, interventions aimed at the removal of excess cytokines may potentially attenuate the acute hyperinflammatory state and may also potentially modulate downstream immunological tolerance.

The CytoSorb hemoadsorption column consists of polystyrene-divinylbenzene copolymer beads coated with polyvinyl-pyrrolidone, which capture inflammatory cytokines with a molecular weight up to approximately 55 kDa by surface adsorption and size exclusion. Although it has been demonstrated to be capable of capturing cytokines in previous in vitro (7) and ex vivo (8) studies, as well as in pigs (9) and humans (10) in vivo, it has not yet been shown to effectively lower circulating plasma cytokine concentrations. For instance, in patients with septic shock, substantial clearance of interleukin (IL)-6 over the CytoSorb adsorber was demonstrated, but plasma concentrations over time were not affected (10). Similarly, plasma concentrations of IL-6 during cardiopulmonary bypass (CPB) in cardiac surgery patients were also not affected by CytoSorb treatment (11).

However, several factors, such as use in unselected patient populations, variability in demographic characteristics, comorbidities, use of medication, time since disease onset, source of the infection, and duration of CytoSorb therapy, may have severely confounded the effects of CytoSorb on plasma cytokine concentrations in these studies. In contrast, the experimental human endotoxemia model, in which healthy volunteers are intravenously challenged with bacterial lipopolysaccharide (LPS), is a highly standardized and reproducible model of systemic inflammation (12). Importantly, this model not only captures many hallmarks of early ‘hyperinflammatory’ sepsis, but also induces a refractory ‘endotoxin-tolerant’ state, which is exemplified by a severely blunted cytokine response upon a second challenge with the same dose of LPS (12, 13). As such, repeated LPS administration can be used as a model for both the hyperinflammatory as well as the immunoparalytic phenotypes of sepsis.

In the present work, we aimed to obtain proof-of-principle for the effects of treatment with CytoSorb hemoperfusion on concentrations of circulating cytokines and development of immunological tolerance during systemic inflammation in healthy volunteers undergoing repeated experimental endotoxemia. Furthermore, we determined cytokine clearance characteristics of the CytoSorb adsorber and its effects on other inflammatory and hemodynamic parameters.

## Materials and methods

### Subjects and ethics

The study protocol was approved by the local ethics committee (CMO Arnhem-Nijmegen; reference nos. NL71293.091 and CMO2019-5730). Twenty-four healthy male volunteers between 18 and 35 years of age were recruited. All subjects provided written informed consent and were included after medical history, physical examination, routine laboratory tests and a 12-lead electrocardiogram revealed no abnormalities. Smoking, use of any medication, previous participation in experimental human endotoxemia, allergies to any of the investigational drugs or signs of acute illness within two weeks prior to the start of the study were considered exclusion criteria. The study was registered at ClinicalTrials.gov (NCT04643639). All study procedures were performed in accordance with the Declaration of Helsinki and its most recent revisions.

### Study design

**Fig. 1:**
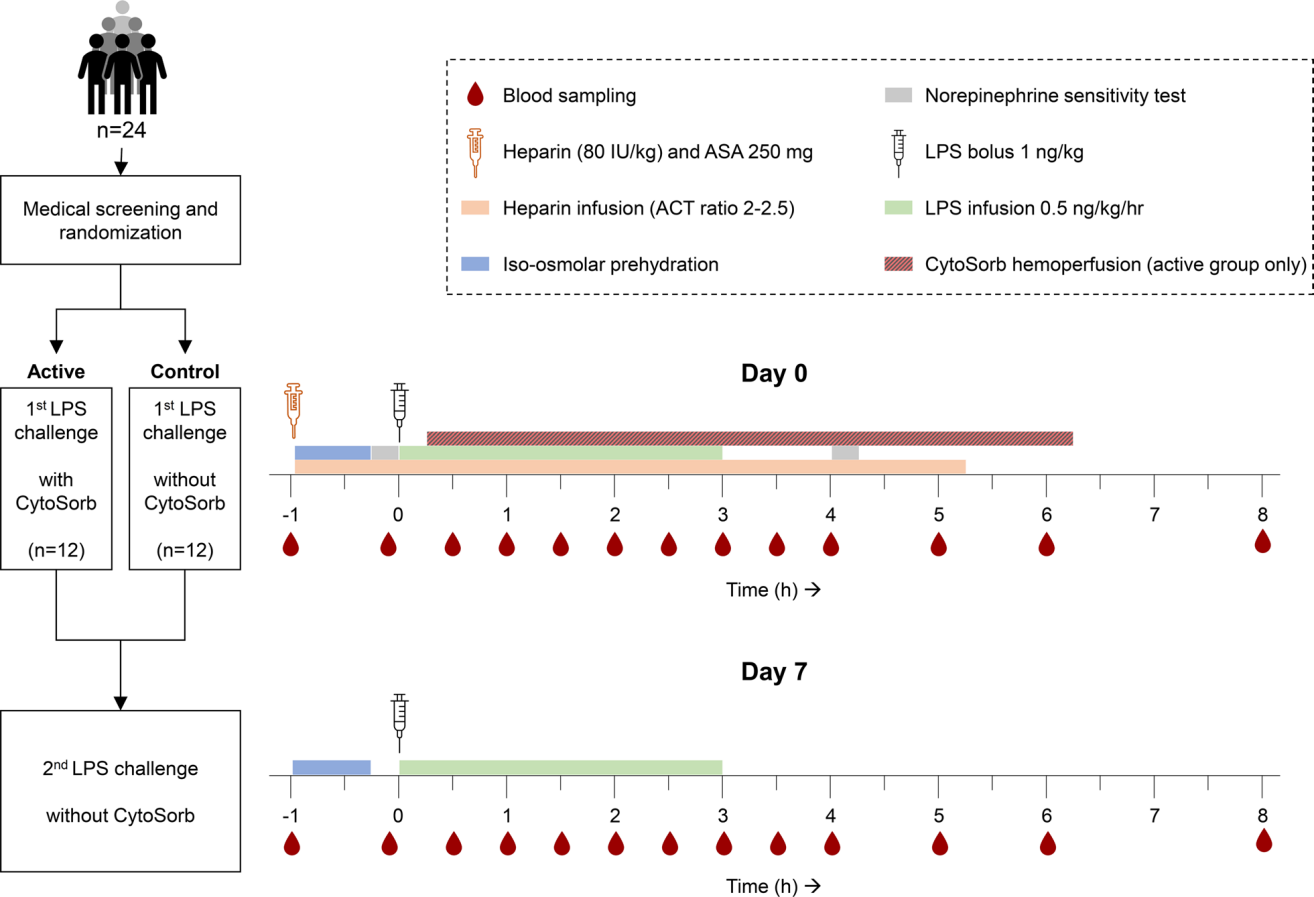
Schematic overview of study procedures. LPS = lipopolysaccharide; ASA = acetylsalicylic acid; ACT = activated clotting time

We performed an open label randomized controlled repeated experimental human endotoxemia study, the design of which is depicted in Fig. 1. After inclusion, all subjects were intravenously challenged twice with the same dose of LPS (details on the experimental human endotoxemia procedure are provided below). The first LPS challenge (on day 0) served to quantify the extent of the primary cytokine response and to induce endotoxin tolerance. The second LPS challenge (on day 7) served to assess the degree of endotoxin tolerance, as reflected by a markedly attenuated cytokine response compared to the first challenge. Subjects were randomized into one of two groups (CytoSorb or control). During the first LPS challenge only, subjects in the CytoSorb group were treated with CytoSorb hemoperfusion for six consecutive hours.

### Experimental human endotoxemia

All endotoxemia-related procedures were performed according to our standard continuous LPS-infusion protocol (12). In short, subjects were admitted to the research unit situated on the Medium Care department of the Radboud university medical center for approximately ten hours. Subjects had to refrain from consumption of alcohol and caffeine (24 h), and food and drinks (12 h) prior to LPS administration. Upon admission on the first LPS challenge day, two antebrachial venous cannulas were placed to allow administration of fluids, study medication and LPS. A radial artery catheter (BD Infusion Therapy Systems, Sandy, Utah, USA) was placed under ultrasound guidance to allow serial blood sampling and continuous hemodynamic monitoring. To reduce the risk of vasovagal responses (14), hydration fluids (2.5% glucose/0.45% sodium chloride) were administered as an initial prehydration bolus of 1.5L in the 45 min prior to LPS administration, and at a rate of 150 mL/hr for the remainder of the experiment. After prehydration, a bodyweight adjusted bolus dose of 1 ng/kg LPS (*E. coli* type O:113, lot no. 94332B1; List Biological Laboratories, Campbell, USA) was administered intravenously, directly followed by continuous infusion of LPS at a rate of 0.5 ng/kg/hr for three hours. Blood samples were serially obtained to construct time-concentration curves of circulating cytokines. Heart rate and intra-arterial blood pressure were measured using a 4-lead electrocardiogram (M50 Monitor, Philips, Eindhoven, the Netherlands) and a radial artery pressure transducer (Edwards Lifesciences, Irvine, California, USA). Hemodynamic parameters were recorded every 5 s using in-house developed software. Every 30 min, body temperature was measured using a tympanic thermometer (FirstTemp Genius 2, Covidien, Dublin, Ireland). Flu-like symptoms (headache, nausea, cold shivers, myalgia and back pain) were scored every 30 min using a numeric six-point Likert scale (0 = no symptoms, 5 = worst ever experienced). A composite score was then calculated as the sum of all previous elements, resulting in a total symptom score ranging from 0 to 25. Furthermore, general malaise was scored on an eleven-point scale (0 = no symptoms, 10 = worst ever experienced).

### CytoSorb hemoperfusion

The CytoSorb 300 mL hemoadsorption device (CytoSorbents Corporation, Princeton, New Jersey, USA) was placed into an extracorporeal circuit on a hemodialysis machine with a dedicated hemoperfusion blood line set (Gambro Prismaflex System and Gambro HP-X line set, Baxter, Deerfield, Illinois, USA). Prior to use, the CytoSorb adsorber was primed with two liters of sodium chloride 0.9% solution as per the manufacturer’s instructions. Next, a hemodialysis catheter (High Flow Double Lumen 13Fr 250 mm, Joline, Hechingen, Germany) was placed in the right femoral vein under ultrasound guidance and local anesthesia, only in subjects of the CytoSorb group. Initial experiments with heparin or bivalirudin anticoagulation monotherapy were terminated prematurely due to clot formation in the extracorporeal circuit, likely related to LPS-induced platelet activation (15). Therefore, 250 mg of acetylsalicylic acid (ASA) in addition to 80 IU/kg of unfractionated heparin was administered intravenously after placement of the catheters. Directly thereafter, heparin was continuously administered until one hour prior to the end of hemoperfusion. Activated clotting time (ACT) was measured every 30–60 min using a point of care analyzer (Hemochron Signature Elite, Werfen, Bedford, USA), and heparin infusion rates were adjusted throughout the experiment to maintain an ACT-ratio between 2 and 2.5 times the baseline value. As ASA and heparin may both have immunomodulatory effects (16, 17), the same anticoagulant regimen was used in the control group to prevent potential confounding related to the administration of these drugs. The goal of this proof-of-principle study was to investigate whether the CytoSorb device is capable of attenuating plasma cytokine concentrations after induction of systemic inflammation. Previous endotoxemia studies have demonstrated that several cytokines already start to increase within 30 min after LPS administration (6). Therefore, CytoSorb hemoperfusion was initiated fifteen minutes after the initial LPS bolus to maximize the chances of demonstrating a treatment effect. To this end, subjects in the CytoSorb group were treated with CytoSorb hemoperfusion for six consecutive hours at a constant blood flow rate of 250 mL/min. After six hours, the hemoperfusion treatment was terminated, the remaining blood in the extracorporeal circuit was returned and subjects were disconnected from the device.

### Vascular reactivity tests

A common feature of systemic inflammation is reduced vascular reactivity, as reflected by an attenuated increase in blood pressure upon norepinephrine administration (18). To assess whether treatment with CytoSorb hemoperfusion affects vascular reactivity, a norepinephrine sensitivity test was performed at baseline and four hours after the LPS bolus administration on the first LPS challenge day. At both timepoints, norepinephrine was continuously administered in increasing dosages (0.05, 0.1 and 0.2 mcg/kg/min) for five minutes per dose. Blood pressure was measured continuously via the radial artery catheter to construct dose–response curves. High-frequency hemodynamic data were captured using specialized software (ICM + , Cambridge Enterprise, Cambridge, UK). Data were synchronized on start-times of norepinephrine infusion, and noise was removed from the high-frequency signal by calculating simple moving averages. Differences in vascular reactivity between and within subjects over time were analyzed by comparing the baseline-corrected area under the curve (AUC) of the blood pressure–time curve.

### Determination of cytokine clearance and elimination by the CytoSorb adsorber

To assess changes in single pass cytokine clearance and elimination over time, paired blood samples from the inlet and outlet ports of the CytoSorb adsorber were serially obtained. Cytokine clearance was calculated according to the following formula:

$${\text{Cl}} = \frac{{\left( {{\text{Ci}} - {\text{Co}}} \right)}}{{{\text{Ci}}}} \times F$$

where Cl = clearance (in mL/min), Ci = inlet plasma cytokine concentration (in pg/mL), Co = outlet plasma cytokine concentration in (pg/mL), and *F* = plasma flow through the adsorber (in mL/min, calculated as blood flow × (1-hematocrit). The cytokine elimination rate (*E*, in pg/min) was calculated as $E = {\text{Cl}} \times {\text{Ci}}$.

### Monocyte HLA-DR expression analysis

Monocyte HLA-DR (mHLA-DR) expression was assessed in ethylenediaminetetraacetic-acid (EDTA)-anticoagulated whole blood using the Anti-HLA-DR/Anti-Monocyte Quantibrite assay (BD Biosciences, San Jose, California, USA) on a CytoFLEX flow cytometer (Beckman Coulter, Indianapolis, Indiana, USA). Flow data were analyzed using Kaluza Software (Beckman Coulter, Indianapolis, Indiana, USA). The number of antibodies bound per cell was calculated by standardizing the geometric mean of monocyte HLA-DR fluorescence intensity (MFI) to BD Quantibrite phycoerythrin (PE) beads (BD Biosciences, San Jose, California, USA). mHLA-DR expression was assessed on both LPS challenge days one hour before, as well as three and six hours after LPS administration.

### Cytokine analysis and hemocytometry

To determine plasma cytokine concentrations, EDTA-anticoagulated blood was centrifuged (10 min, 2000 g, 4 °C) directly after withdrawal, and plasma was stored at − 80 °C until analysis. Concentrations of tumor necrosis factor (TNF), interleukin (IL)-6, IL-8, IL-10, macrophage inflammatory protein (MIP)-1*α*, monocyte chemoattractant protein (MCP)-1, granulocyte colony stimulating factor (G-CSF) and interferon-*γ*-induced protein (IP)-10 were determined batch-wise using a simultaneous Luminex assay (Milliplex, Millipore, Billerica, USA) as per the manufacturer’s instructions. Hemocytometry parameters were measured in EDTA-anticoagulated whole blood using a fully automated hematology analyzer (Sysmex XN-1000, Sysmex Corporation, Kobe, Japan).

### Statistical analysis

Distribution of data was assessed for normality based on histogram plots and Shapiro–Wilk’s test, and data with a non-Gaussian distribution were log-transformed prior to statistical testing if a nonparametric equivalent test was not available. Normally distributed data are presented as mean ± standard error of the mean, whereas nonparametric data are presented as median and interquartile range. The AUC of the plasma cytokine concentration–time curve was calculated as an integral measure of cytokine responses over time for each LPS challenge day. Within-group differences in cytokine concentrations, body temperature, symptom score, hemodynamic, and hemocytometry parameters over time were analyzed using one-way repeated measures analysis of variance (RM-ANOVA), whereas between-group differences over time were analyzed using two-way RM-ANOVA (time × group interaction term). All data analysis and visualization were performed using R version 4.1.3 (The R Foundation for Statistical Computing, Vienna, Austria).

## Results

Twenty-four healthy male volunteers were included in this study. Baseline demographic characteristics are listed in Table 1. All procedures and symptoms associated with the experimental endotoxemia and hemoperfusion procedures were well tolerated by all subjects, and no serious adverse events occurred during the study.

**Table 1:** Baseline demographic characteristics of the two study groups

Variable	Control group (*n* = 12)	CytoSorb group (*n* = 12)
Age (y)	22.7 ± 1.0	22.2 ± 0.9
Height (cm)	182 ± 1.7	182 ± 1.2
Weight (kg)	76.9 ± 2.7	77.9 ± 2.6
BMI (kg/m^2^)	23.3 ± 0.8	23.5 ± 0.8
Hemoglobin (mmol/L)	9.1 ± 0.2	9.4 ± 0.1
Creatinine (μmol/L)	79 ± 3	78 ± 4
Leukocyte count (10^9^/L)	5.6 ± 0.3	6.3 ± 0.5
Platelet count (10^9^/L)	233 ± 15	238 ± 17

Data displayed as mean ± standard error of the mean

*BMI* Body mass index

### Effects of CytoSorb hemoperfusion on plasma cytokine concentrations

**Fig. 2:**
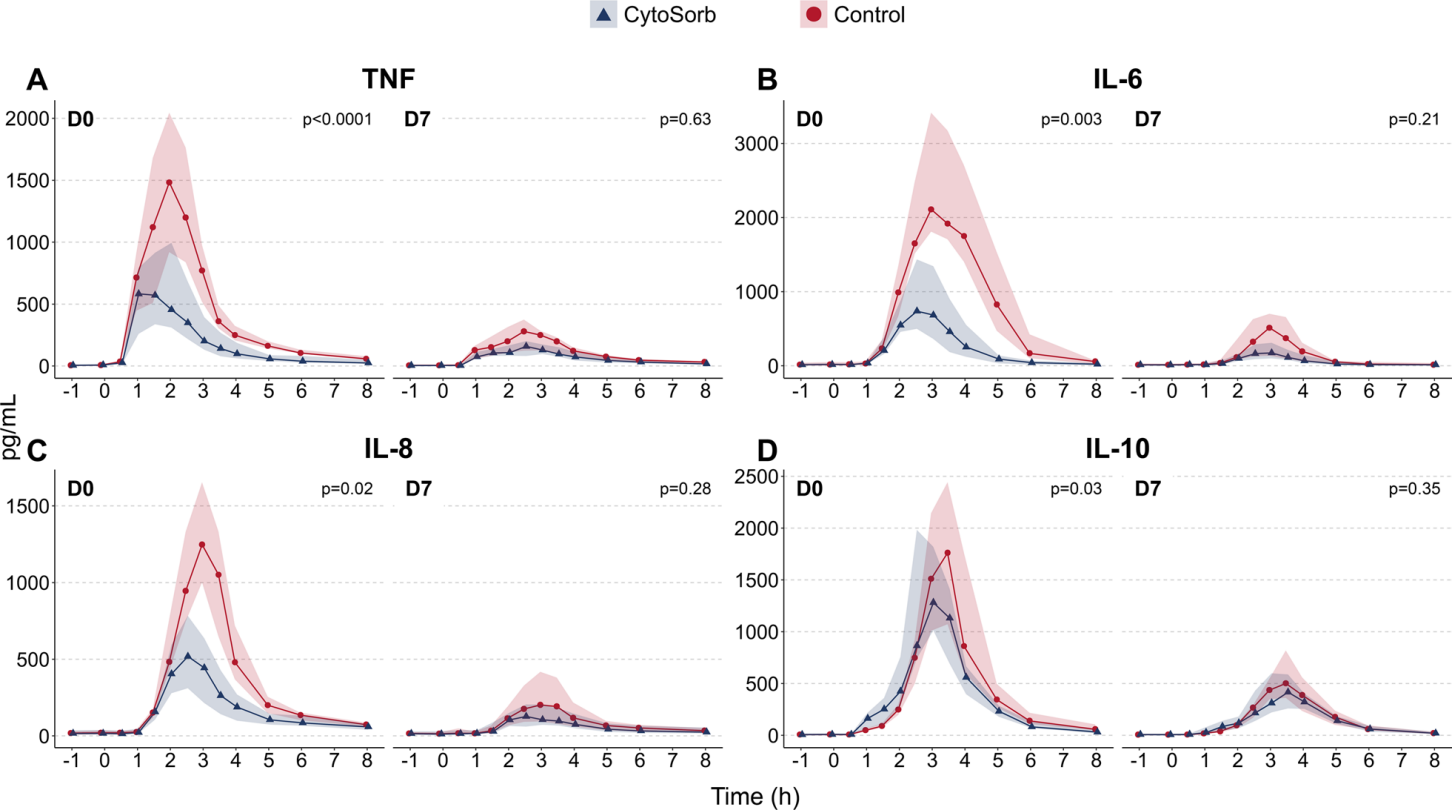
Plasma concentrations of **A** TNF,** B** IL-6,** C** IL-8 and** D** IL-10 during the first (D0) and second (D7) LPS challenge day. Data are displayed as median (line) and interquartile range (shaded area). *P* values were computed using two-way repeated measures analysis of variance (time × group interaction term). D0 = day 0, D7 = day 7, TNF = tumor necrosis factor, IL = interleukin

Intravenous administration of LPS resulted in a profound but transient increase in concentrations of all measured cytokines on both LPS challenge days (Fig. 2 and Additional file 1: Fig. S1). In subjects of the CytoSorb group, the increase in plasma concentrations of cytokines upon the first LPS challenge was significantly attenuated compared to the control group: TNF (median AUC − 58%, *p* < 0.0001), IL-6 ( − 71%, *p* = 0.003), IL-8 ( − 48%, *p* = 0.02), IL-10 ( − 26%, *p* = 0.03), MCP-1 ( − 34%, *p* = 0.02), and MIP-1*α* ( − 39%, *p* = 0.006), while a trend toward less pronounced G-CSF concentrations was observed ( − 47%, *p* = 0.054, Fig. 2 and Additional file 1: Fig. S1). Interestingly, plasma concentrations of the chemokine IP-10 were not significantly affected during the first challenge (+ 25%, *p* = 0.15, Additional file 1: Fig. S1A). Upon the second LPS challenge seven days later, a severely blunted response was observed in both groups, as reflected by significantly attenuated plasma concentrations of all cytokines as compared to the first challenge (all *p* < 0.0001), indicative of profound endotoxin tolerance (Fig. 2 and Additional file 1: Fig. S1). However, no significant between-group differences in plasma concentrations of any of the measured cytokines were observed during the second challenge (Fig. 2 and Additional file 1: Fig. S1). Furthermore, no significant reduction in mHLA-DR expression, a well-established marker of immunological tolerance, was observed during the first LPS challenge in either group, whereas the decrease in mHLA-DR expression during the second LPS challenge was similar in both groups (Additional file 2: Fig. S2).

### Cytokine clearance by the CytoSorb adsorber

**Fig. 3:**
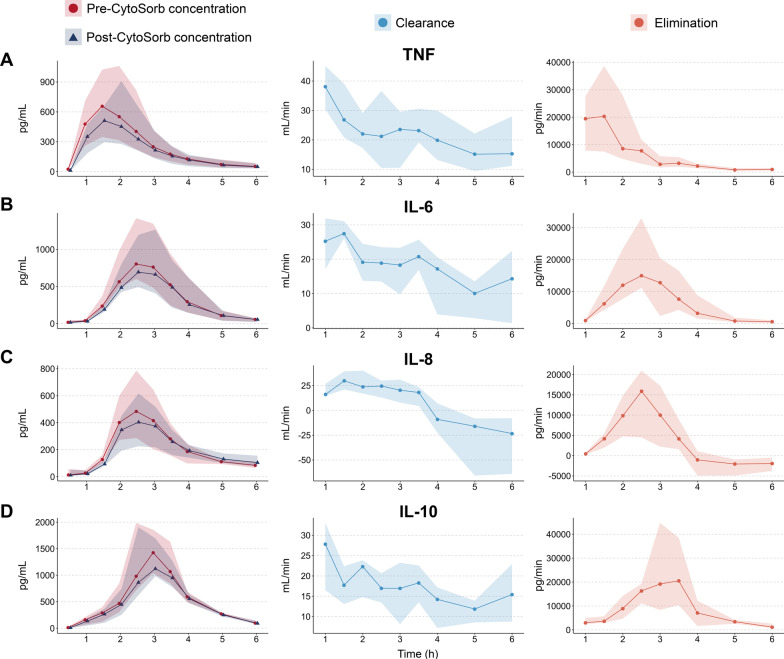
Plasma concentrations measured at the inlet and outlet ports of the adsorber (left panels), clearance rates (center panels) and elimination rates (right panels) of** A** TNF,** B** IL-6,** C** IL-8 and** D** IL-10 during the first LPS challenge day in the CytoSorb group. Data are displayed as median (line) and interquartile range (shaded area). TNF = tumor necrosis factor, IL = interleukin

Cytokine clearance and elimination rates are displayed in Fig. 3 and Additional file 3: Fig. S3. Although all measured cytokines were removed from the circulation by the adsorber to some degree, some were cleared more effectively than others with median clearance rates throughout the experiment varying from 26 (21﻿–28) mL/min for TNF to 7 (2﻿–21) mL/min for G-CSF (Fig. 3 and Additional file 3: Fig. S3). Clearance rates showed a declining trend over time, possibly due to saturation of the adsorber. For example, between one and six hours after the LPS bolus administration, median clearance rates of IP-10 attenuated by 83%, whereas IL-6 clearance rates decreased by 43%. For some cytokines, such as IL-8 and MIP-1*α*, clearance and elimination rates became negative after several hours, indicating that the adsorber releases cytokines back into the circulation when fully saturated, although the absolute amounts were small and the impact on plasma concentrations therefore negligible (Fig. 3 and Additional file 3: Fig. S3).

### Effect of CytoSorb hemoperfusion on hemodynamic parameters and vascular reactivity

**Fig. 4:**
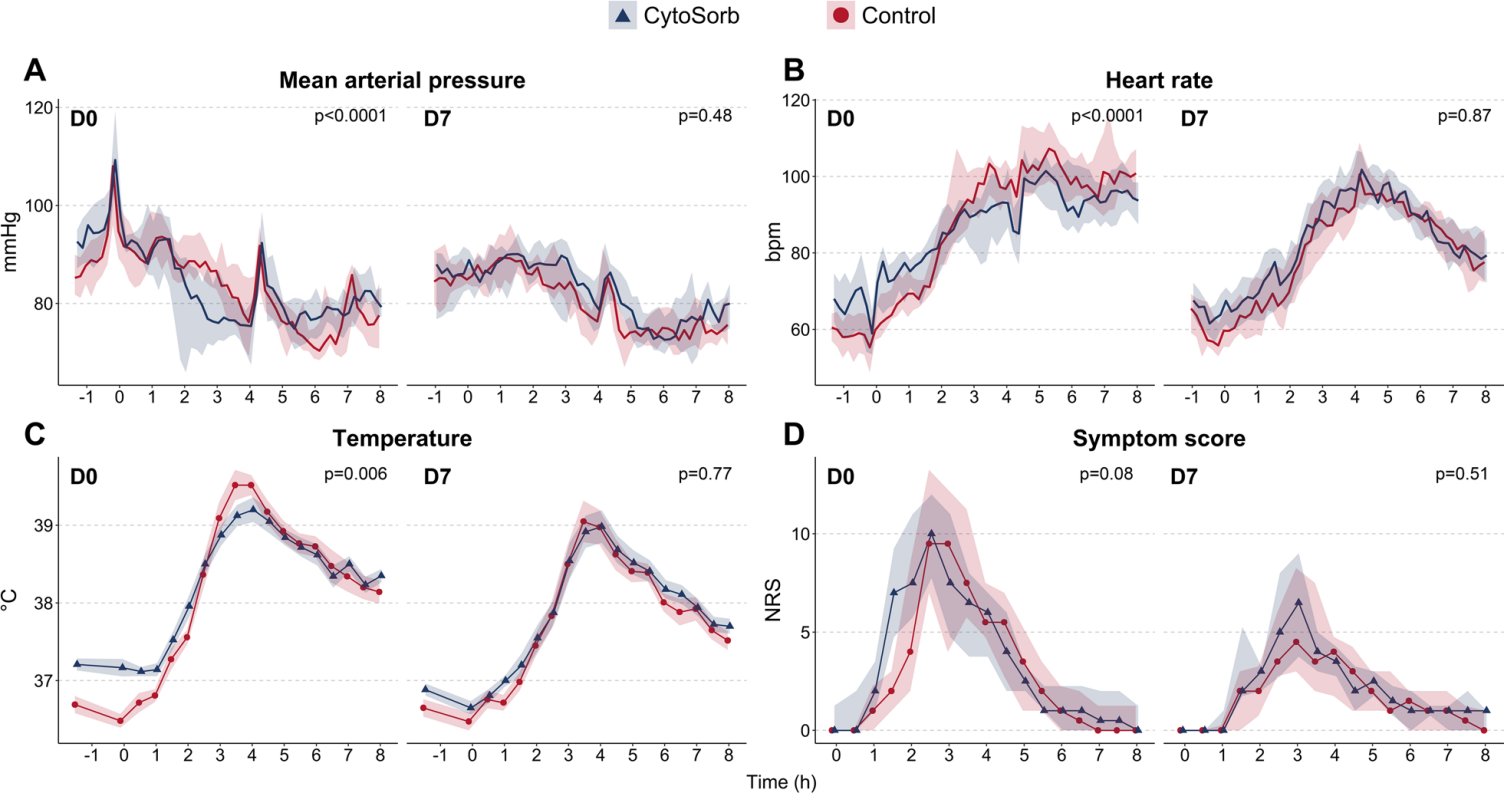
Clinical parameters during the first (D0) and second (D7) LPS challenge. **A** Mean arterial pressure, **B** heart rate, **C** temperature, and **D** symptom score. Data in panels **A**, **B**, and **D** are displayed as median (line) and interquartile range (shaded area), whereas data in panel **C** are displayed as mean and standard error of the mean. *P* values were computed using two-way repeated measures analysis of variance (time × group interaction term). D0 = day 0, D7 = day 7, NRS = numeric rating scale

Administration of LPS resulted in a decrease in blood pressure and a compensatory increase in heart rate in both groups during both LPS challenge days, albeit to a less extent upon the second challenge (Fig. 4AB). In subjects treated with CytoSorb during the first LPS challenge, these effects occurred earlier, but were less sustained compared to the control group (both *p* < 0.0001). Treatment with CytoSorb during the first LPS challenge did not influence the decrease in blood pressure observed during the second LPS challenge (Fig. 4AB). The increase in blood pressure induced by intravenous administration of increasing dosages of norepinephrine at baseline (just prior to the first LPS administration) did not differ significantly from the response four hours later in either of the two groups (Additional file 4: Fig. S4A). This indicates that experimental human endotoxemia did not significantly attenuate norepinephrine sensitivity. Although subjects in the CytoSorb group demonstrated a significantly more pronounced increase in blood pressure than subjects of the control group upon administration of norepinephrine prior to the LPS challenge, the magnitude of this difference did not change upon administration of norepinephrine four hours after the LPS challenge (*p* < 0.01 at both timepoints, Additional file 4: Fig. S4B).

### Effects of CytoSorb hemoperfusion on body temperature and symptom scores

LPS administration induced a substantial increase in body temperature in both groups during both LPS challenge days (Fig. 4C). Prior to LPS administration on the first LPS challenge day, body temperature was higher in the CytoSorb group. As this measurement was obtained shortly after placement of the dialysis catheter, this difference is probably caused by the fact that subjects in the CytoSorb group were covered by a sterile cloth for 20–30 min during the cannulation procedure, whereas subjects in the control group (in whom no dialysis catheter was placed) were not covered. Nevertheless, the LPS-induced increase in body temperature during the first challenge was less pronounced in subjects treated with CytoSorb (*p* = 0.006), whereas no significant group differences in body temperature were observed upon the second LPS challenge (*p* = 0.77, Fig. 4C). Although the onset of the LPS-induced increase in the composite score of flu-like symptoms appeared to be earlier in subjects in the CytoSorb group, this difference did not reach statistical significance (*p* = 0.08, Fig. 4D). Further exploration of individual symptoms, displayed in Additional file 5: Fig. S5, revealed that the sensation of shivering was experienced earlier and more pronounced in the CytoSorb group (*p* < 0.0001, Additional file 5: Fig. S5E). In line, the sensation of general malaise was also experienced earlier and more severely in subjects treated with CytoSorb during the first LPS challenge (*p* = 0.004, Additional file 5: Fig. S5F). Treatment with CytoSorb did not influence symptom score severity during the second LPS challenge, with exception of a slightly more pronounced sensation of myalgia in the CytoSorb group (*p* = 0.004, Additional file 5: Fig. S5C).

### Effects of CytoSorb hemoperfusion on hemocytometry parameters

Hemocytometry data during both LPS challenges are displayed in Additional file 6: Fig. S6. Administration of LPS induced a minor decrease in platelet counts, and this effect was slightly more pronounced in subjects treated with CytoSorb hemoperfusion during the first LPS challenge (*p* < 0.001, Additional file 6: Fig. S6B). Similarly, the LPS-induced increase in total leukocyte and neutrophil counts was also more apparent (*p* = 0.006 and *p* = 0.04, respectively, Additional file 6: Fig. S6C, D) in the CytoSorb group during the first challenge, whereas the reduction in lymphocyte counts was less pronounced (*p* < 0.0001, Additional file 6: Fig. S6E). Although treatment with CytoSorb did not affect the disappearance of monocytes from the circulation within 1–2 h after LPS administration, the number of circulating monocytes returned to baseline concentrations significantly earlier in the CytoSorb group during the first LPS challenge (*p* = 0.002, Additional file 6: Fig. S6F). During the second LPS challenge, no differences in any of the measured hemocytometry parameters were observed between the two groups.

## Discussion

Although the CytoSorb hemoadsorption device has been demonstrated to be capable of capturing inflammatory cytokines in previous in vitro, ex vivo and in vivo studies, it has hitherto not yet been demonstrated to effectively lower concentrations of circulating cytokines in a randomized study. Possibly, results from these previous studies may have been confounded by several factors, such as suboptimal treatment protocols and/or unselected patient populations with relatively less elevated cytokine concentrations and a high degree of patient heterogeneity. Moreover, any potential late effects of CytoSorb treatment on the induction of immunological tolerance remained uninvestigated as of yet. Herein, we provide proof-of-principle that treatment with CytoSorb hemoperfusion markedly attenuates plasma concentrations of circulating cytokines in a highly reproducible model of pronounced systemic inflammation, whereas induction of endotoxin tolerance is not affected.

Several previous studies have investigated the cytokine adsorption capacity of the CytoSorb cartridge and its effects on plasma cytokine concentrations in vivo. For instance, substantial single pass IL-6 elimination has been described both in a pig model of severe smoke and burn injury (9), as well as in 100 mechanically ventilated sepsis patients (10). Nevertheless, concentrations of circulating cytokines were not affected in either study (9, 10). Similarly, no significant effect of CytoSorb treatment on cytokine concentrations was found in 21 patients with post-cardiac arrest syndrome (19). Importantly, as no form of patient enrichment was applied in the patient studies, cytokine concentrations were relatively low, thereby decreasing the a priori chances of demonstrating a therapeutic effect of CytoSorb treatment (10, 19). Moreover, in several of these studies, CytoSorb therapy was applied as a single treatment of six hours per day (9, 10), and it is therefore plausible that the majority of cytokines were produced outside this therapeutic window. This might also explain why CytoSorb therapy applied during cardiopulmonary bypass did not affect plasma concentrations of IL-6 in 37 cardiac surgery patients (11), as cytokine concentrations are known to peak several hours after termination of CPB in these patients (11, 20), and CytoSorb therapy in this particular study was ceased at the end of CPB (11). Given the pronounced effect on plasma cytokine concentrations in the present work, it appears plausible that these studies might have yielded different results if patient groups with a more pronounced cytokine response were selected and received more prolonged or continuous hemoadsorption treatments.

A clinically relevant result of our study is the finding that cytokine clearance rates decrease over time. Although some cytokines were affected more than others, this indicates that CytoSorb treatment might become less effective as the device saturates. This is further exemplified by the fact that clearance and elimination rates of several cytokines (MIP-1*α* and IL-8 in particular) even became negative, indicating that small amounts of cytokines can be released back into the circulation. However, since circulating cytokine concentrations during these later timepoints were low, the absolute amount of cytokines released back into the circulation was limited and did not relevantly impact plasma concentrations. Although this phenomenon, also referred to as desorption, has been described before during CytoSorb therapy for beta-lactam antibiotics (21) and 3,4-methylenedioxymethamphetamine (MDMA, ‘ecstasy’) (22), it had not yet been demonstrated for cytokines. As such, decreased clearance rates over time and cytokine desorption must be taken into account when considering exchange intervals of the CytoSorb cartridge for future trials or in clinical practice.

The removal of cytokines from the circulation by CytoSorb therapy during the first LPS challenge did not alter the immunological response during the second LPS challenge. Moreover, kinetics of mHLA-DR, one of the most commonly used markers of immune (dys)function in critically ill patients (23, 24), were also not affected by CytoSorb therapy. This is the first time that the effect of CytoSorb treatment on the development of immunological tolerance was assessed and our findings appear to be in contrast with a previous study that demonstrated that treatment with polymyxin B hemoperfusion, a blood purification technique which selectively removes bacterial LPS from the circulation (7), improves mHLA-DR expression in severe sepsis patients (25). However, it must be noted that in our study mHLA-DR expression did not decrease upon the first LPS challenge that may have precluded the demonstration of a potential downstream therapeutic effect of CytoSorb. Since mHLA-DR expression was significantly attenuated in response to a second LPS challenge also in previous endotoxemia studies (26), it appears likely that the administration of ASA may have interfered with expression levels during the first LPS challenge, which has been described previously (16). Nonetheless, the similar degree of endotoxin tolerance observed upon the second LPS challenge in both groups suggests that CytoSorb treatment does not impact subsequent immunological competence.

Based on the finding that plasma cytokine concentrations are attenuated by CytoSorb treatment, while the development of immunological tolerance is not, we speculate that tolerance must be rapidly induced in tissue-resident immune cells by locally produced cytokines before these cytokines even reach the circulation. Earlier work from our group demonstrated a complete lack of correlation between circulating cytokine concentrations and ex vivo cytokine production capacity of immune cells (6, 13), thereby implying that the contribution of circulating leukocytes to blood cytokine concentrations and immunological tolerance following LPS administration is very limited, also illustrates the relevance of tissue-resident macrophages. However, it needs to be acknowledged that a role for endothelial cells cannot be ruled out, as these are known cytokine producers (27, 28) and may be rapidly reprogrammed to a tolerant phenotype upon contact with cytokines that enter the circulation from the tissues.

Another common hallmark of severe systemic inflammation is hemodynamic instability and attenuated sensitivity to vasopressors (18). Moreover, several case series and retrospective studies suggest that CytoSorb treatment might result in swift hemodynamic stabilization and a reduction in vasopressor requirements (29–31). Therefore, we aimed to investigate whether CytoSorb treatment improved vascular reactivity to norepinephrine administration. Unfortunately, as administration of LPS did not significantly attenuate norepinephrine sensitivity in either of the groups, a treatment effect of the CytoSorb adsorber on inflammation-induced vasopressor sensitivity could not be established and this question remains unanswered. Nevertheless, one could speculate that the observed decrease in blood pressure in response to LPS administration stabilized earlier in the CytoSorb group might still hint toward beneficial hemodynamic effects of CytoSorb treatment.

Combined, our data indicate that CytoSorb therapy has the potential to attenuate circulating cytokine concentrations in systemically inflamed patients. From a clinical perspective, this also raises the question if CytoSorb therapy might specifically be beneficial in selected patient subgroups with proven hyperinflammation. Our results also suggest that the acute cytokine reductions observed during therapy do not have a late impact on patients’ immunological phenotype. Accordingly, this suggests that clinical effects of cytokine removal (i.e., hemodynamic stabilization) likely would be apparent early. In future trials, the use of enrichment strategies (32, 33), such as measurement of cytokines or ferritin (34) concentrations prior to inclusion, might necessarily demonstrate therapeutic efficacy. Moreover, future trialists and clinicians should make sure to implement timely renewal of the adsorbers during therapy to ensure effective ongoing removal of inflammatory cytokines. Finally, the absence of any device-related adverse events in both our study and also in other available published reports suggests that the safety profile of CytoSorb therapy is favorable.

Strengths of this study are the highly standardized and reproducible nature of the experimental human endotoxemia model. In contrast to clinical trials in sepsis or cardiac surgery patients, our study population is highly selected and homogeneous, and the magnitude and timing of the immunological insult is identical in each subject. Moreover, as the same anticoagulant regimen was used in both the CytoSorb and the control group, the results of this study are unlikely to be influenced by such confounding factors. Clearly, some limitations of this study also need to be addressed. For instance, since no sham-device was available, subjects in the control group, while they did receive the same anticoagulation treatment, did not receive any form of extracorporeal circulation, and the trial was therefore open label. However, since the primary endpoint of this study was cytokine concentrations, the potential unwanted consequences of the unblinded design appear very limited. Although one could argue that the extracorporeal circuit itself may have immunomodulatory effects, the aim of this study was to investigate the effect of CytoSorb hemoperfusion compared to no intervention. Since extracorporeal circulation is an integral part of CytoSorb treatment, the conclusions of this study remain valid nevertheless. Moreover, although the homogeneous population is a benefit for this proof-of-principle study in terms of confounding factors, it clearly also represents a limitation since results of this study cannot be extrapolated to populations with different demographic characteristics and/or comorbidities. Also related to the proof-of-principle nature of this translational study, it cannot address potential clinical benefit in (subgroups of) sepsis patients, which clinical future studies should address.

In conclusion, CytoSorb hemoperfusion markedly attenuates circulating cytokine concentrations during systemic inflammation in humans in vivo, while induction of immunological tolerance is not affected. Potential therapeutic efficacy in the clinical setting should be further investigated in trials employing enrichment strategies to include patients with hyperinflammation. Moreover, continuous treatment regimens and timely renewal of the CytoSorb cartridge might further increase chances of demonstrating a clinical effect. If our findings can be confirmed in such patient studies, CytoSorb therapy could be of clinical benefit in conditions characterized by excessive cytokine release.

### Supplementary Information

**Additional file 1: Fig. S1**. Plasma concentrations of various cytokines during the first (D0) and second (D7) LPS challenge day. Data are displayed as median (line) and interquartile range (shaded area). *P* values were computed using two-way repeated measures analysis of variance (time × group interaction term). D0 = day 0, D7 = day 7, IP = interferon-*γ*-induced protein, G-CSF = granulocyte colony stimulating factor, MCP = monocyte chemoattractant protein, MIP = macrophage inflammatory protein.

**Additional file 2: Fig. S2.** Human leukocyte antigen (HLA)-DR expression on monocytes during the first (D0) and second (D7) LPS challenge day. Data are displayed as mean (line) and standard error of the mean (shaded area). *P* values were computed using two-way repeated measures analysis of variance (time × group interaction term).

**Additional file 3: Fig. S3.** Plasma concentrations measured in samples obtained at the inlet and outlet ports of the adsorber (left panels), clearance rates (center panels) and elimination rates (right panels) of various cytokines during the first LPS challenge day in the CytoSorb group. Data are displayed as median (line) and interquartile range (shaded area).

**Additional file 4: Fig. S4.** Vascular reactivity tests during the first LPS challenge day. Data are displayed as mean (line) and standard error of the mean (shaded area). *P* values in panel A were computed using Wilcoxon’s signed rank test, whereas *p* values in panel B were computed using a Mann–Whitney U test. NE = norepinephrine.

**Additional file 5: Fig. S5.** Scores of various flu-like symptoms during the first (D0) and second (D7) LPS challenge day. Data are displayed as median (line) and interquartile range (shaded area). *P* values were computed using two-way repeated measures analysis of variance (time × group interaction term). D0 = day 0, D7 = day 7.

**Additional file 6: Fig. S6.** Hemocytometry parameters during the first (D0) and second (D7) LPS challenge day. Data are displayed as median (line) and interquartile range (shaded area). *P* values were computed using two-way repeated measures analysis of variance (time × group interaction term). D0 = day 0, D7 = day 7.

## Data Availability

Individual data will be provided upon reasonable request to the corresponding author.
