# Supplementary figures and images for: CytoSorb hemoperfusion markedly attenuates circulating cytokine concentrations during systemic inflammation in humans in vivo
Source: Crit Care. 2023 Mar 21;27:117. doi: 10.1186/s13054-023-04391-z (PMC10029173; doi:10.1186/s13054-023-04391-z)

▲ CytoSorb

● Control

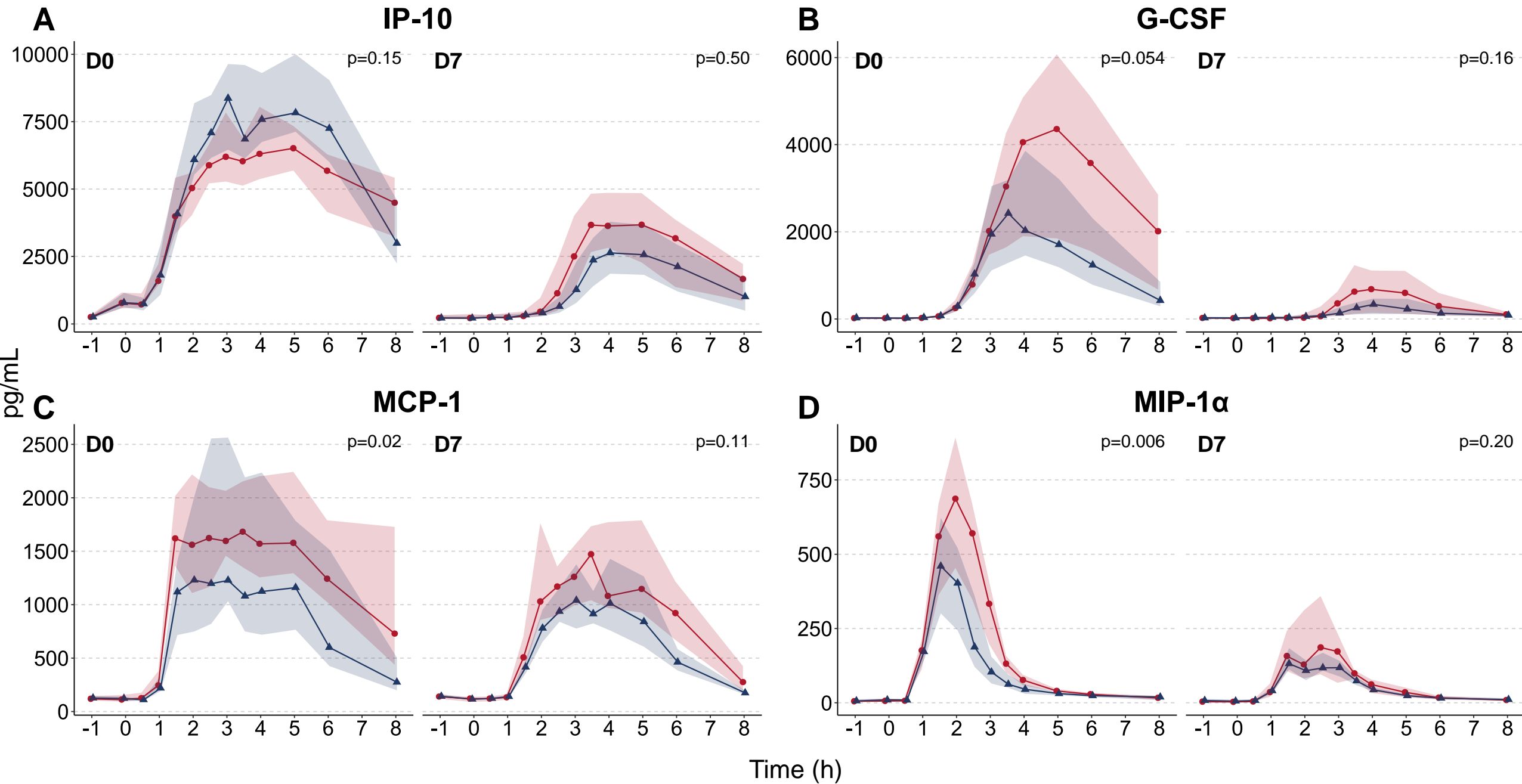

Supplement: Supplementary file 1 — Additional file 1: Fig. S1. Plasma concentrations of various cytokines during the first (D0) and second (D7) LPS challenge day. Data are displayed as median (line) and interquartile range (shaded area). P values were computed using two-way repeated measures analysis of variance (time × group interaction term). D0 = day 0, D7 = day 7, IP = interferon-γ-induced protein, G-CSF = granulocyte colony stimulating factor, MCP = monocyte chemoattractant protein, MIP = macrophage inflammatory protein. [file 13054_2023_4391_MOESM1_ESM.pdf]

▲ CytoSorb

● Control

## HLA-DR expression

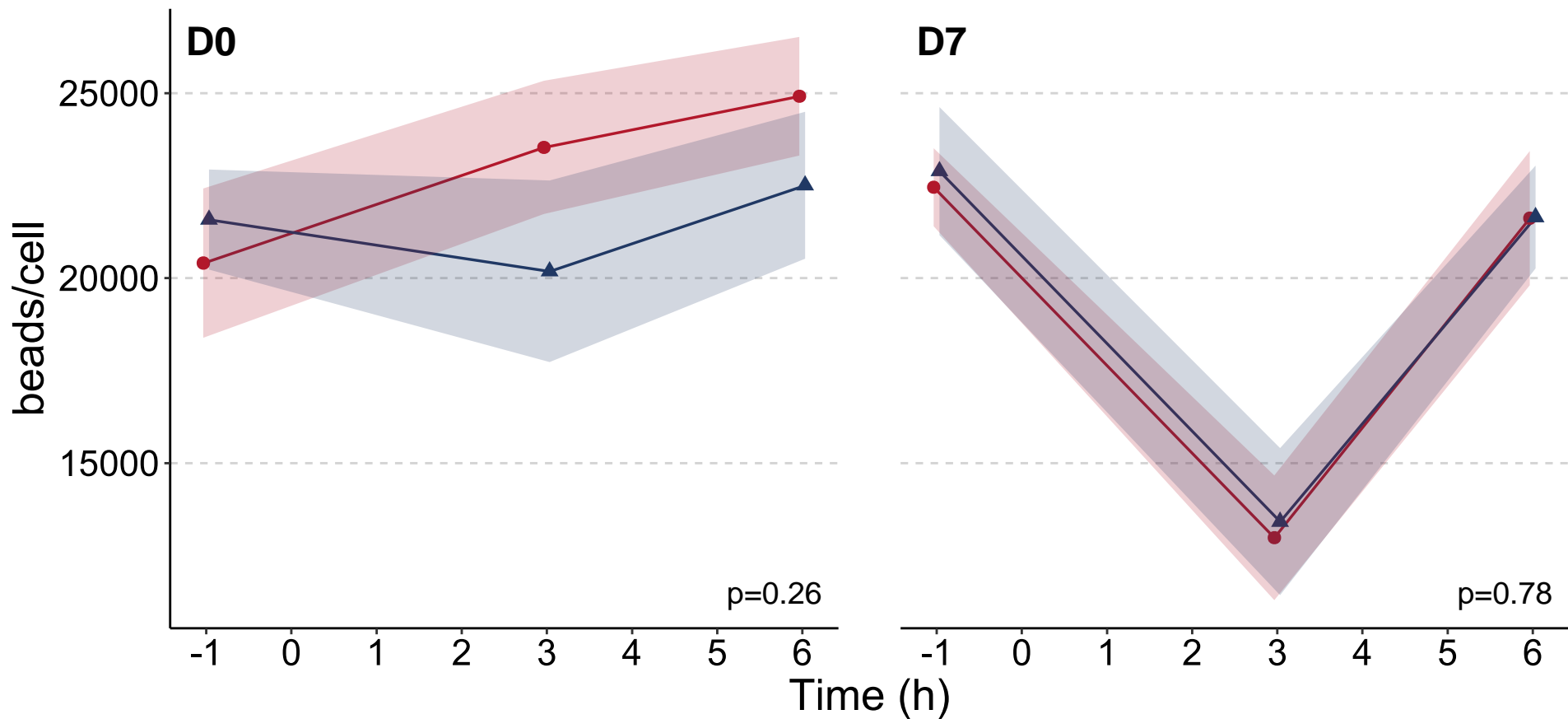

Supplement: Supplementary file 2 — Additional file 2: Fig. S2. Human leukocyte antigen (HLA)-DR expression on monocytes during the first (D0) and second (D7) LPS challenge day. Data are displayed as mean (line) and standard error of the mean (shaded area). P values were computed using two-way repeated measures analysis of variance (time × group interaction term). [file 13054_2023_4391_MOESM2_ESM.pdf]

● Pre-CytoSorb concentration  
▲ Post-CytoSorb concentration

● Clearance

● Elimination

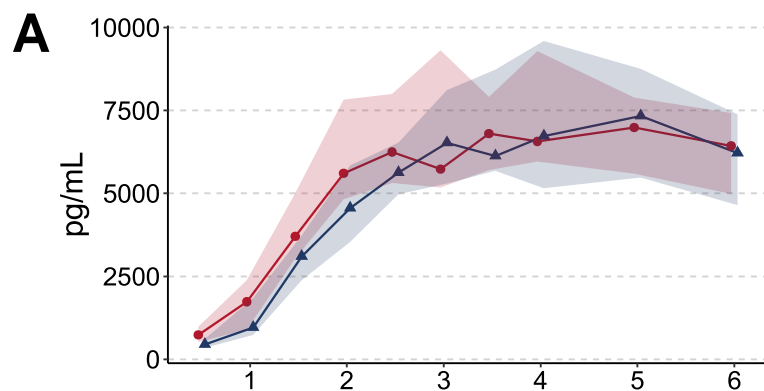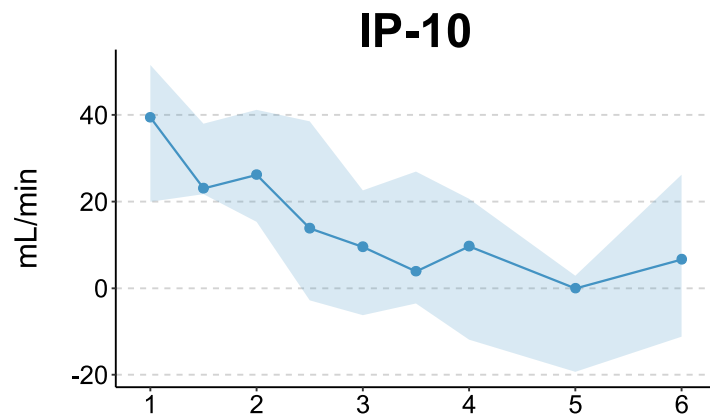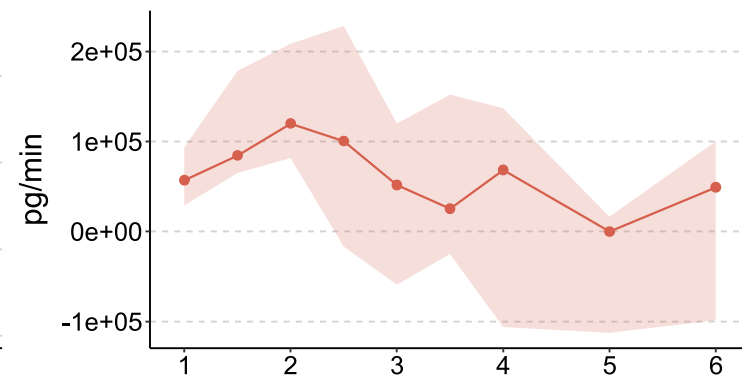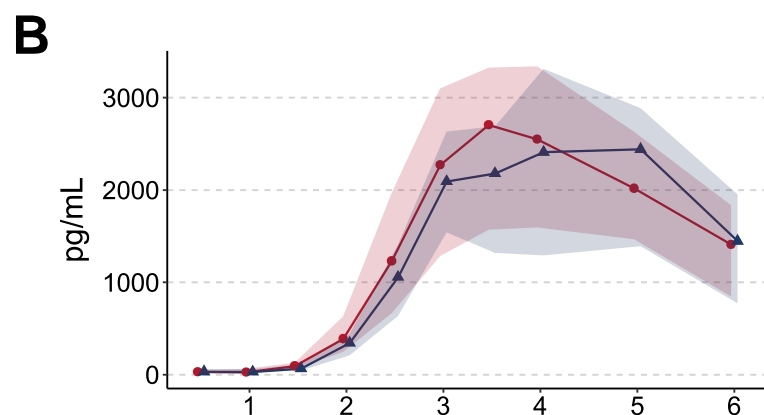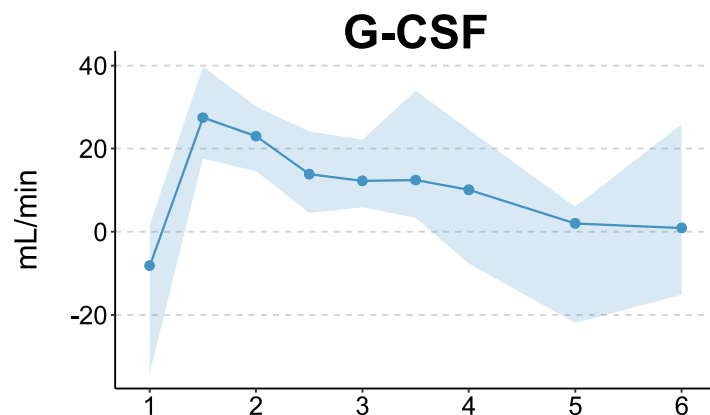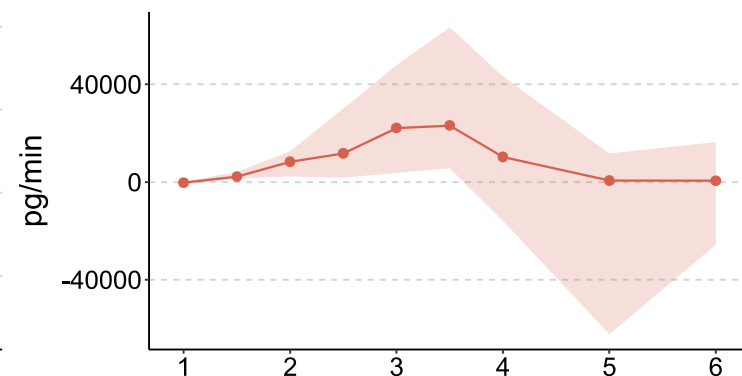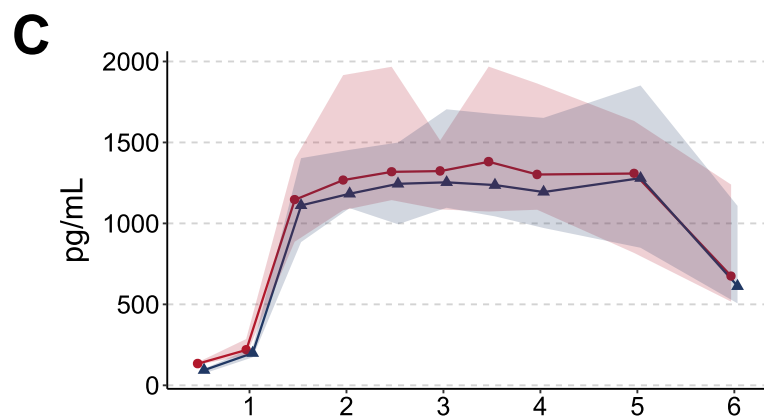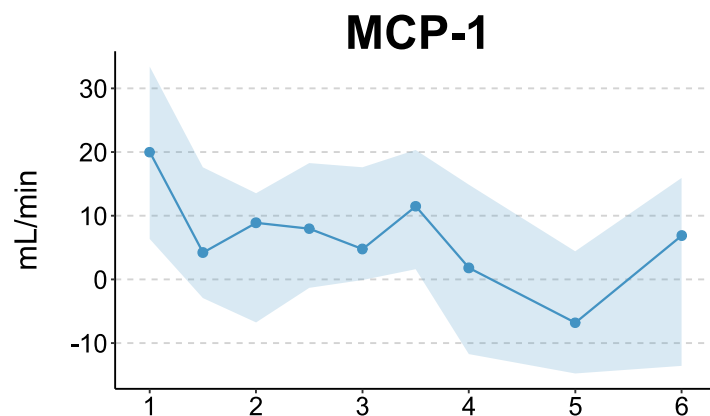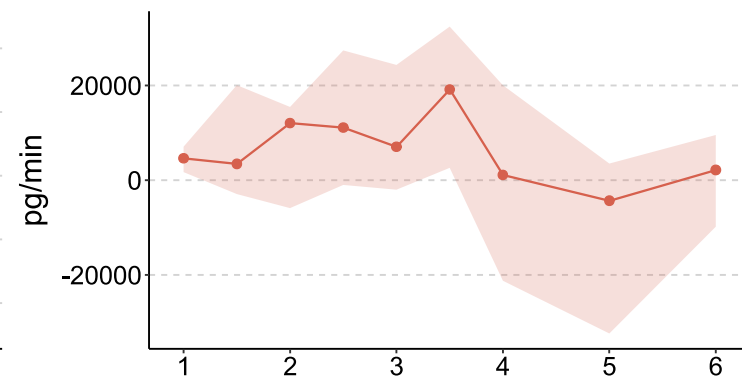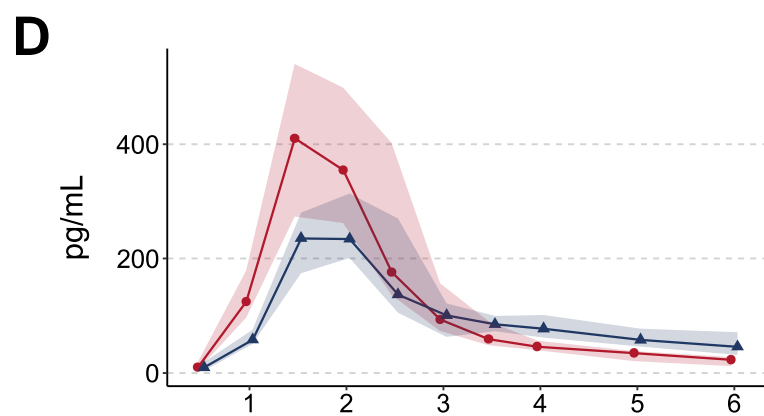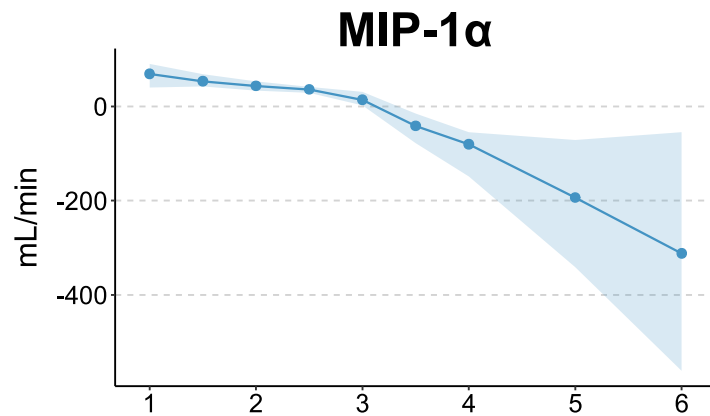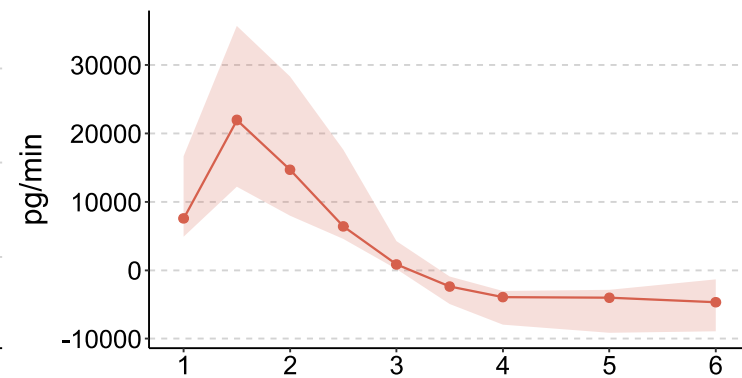

Time (h)

Supplement: Supplementary file 3 — Additional file 3: Fig. S3. Plasma concentrations measured in samples obtained at the inlet and outlet ports of the adsorber (left panels), clearance rates (center panels) and elimination rates (right panels) of various cytokines during the first LPS challenge day in the CytoSorb group. Data are displayed as median (line) and interquartile range (shaded area). [file 13054_2023_4391_MOESM3_ESM.pdf]

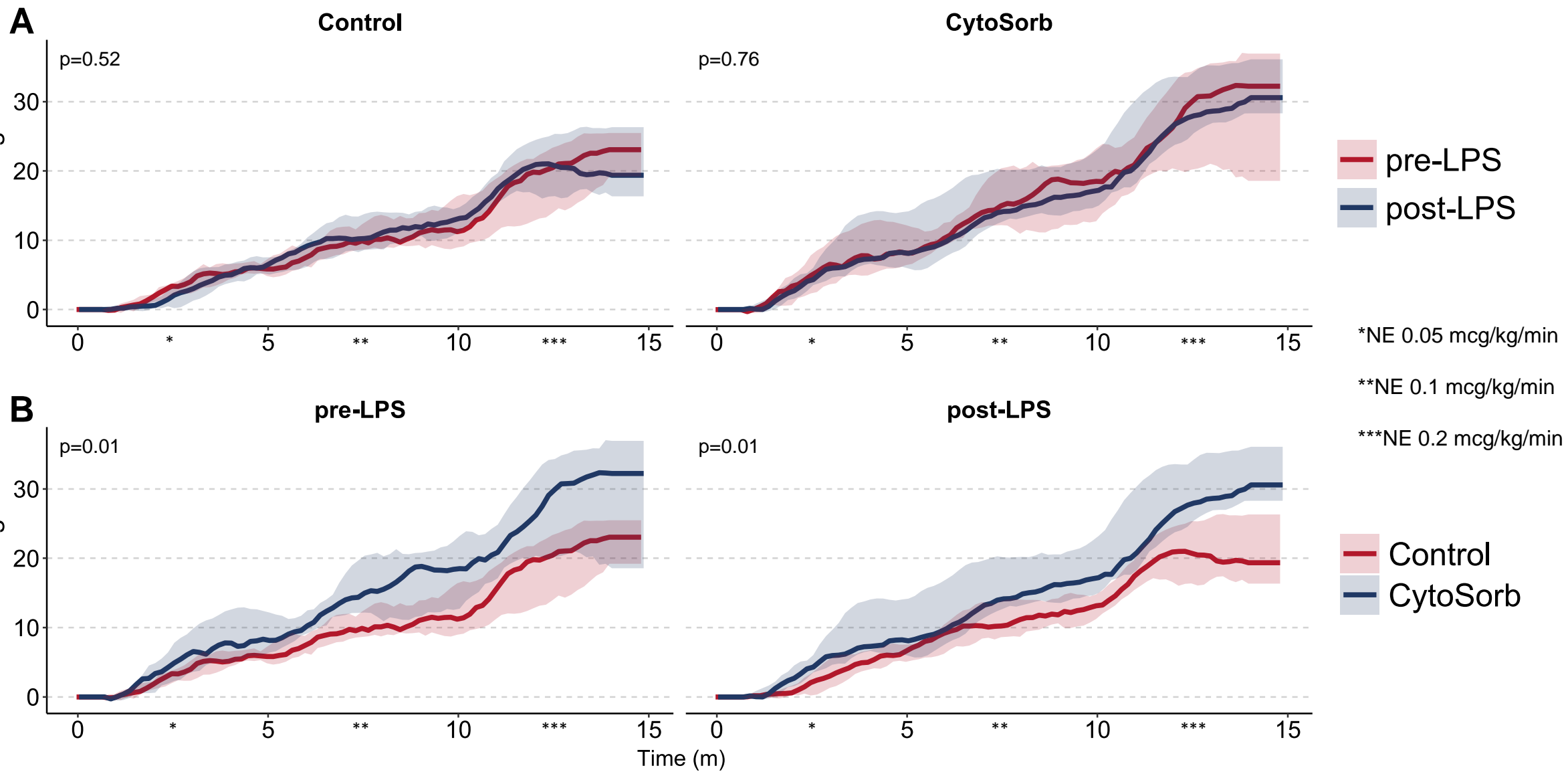

Supplement: Supplementary file 4 — Additional file 4: Fig. S4. Vascular reactivity tests during the first LPS challenge day. Data are displayed as mean (line) and standard error of the mean (shaded area). P values in panel A were computed using Wilcoxon’s signed rank test, whereas p values in panel B were computed using a Mann–Whitney U test. NE = norepinephrine. [file 13054_2023_4391_MOESM4_ESM.pdf]

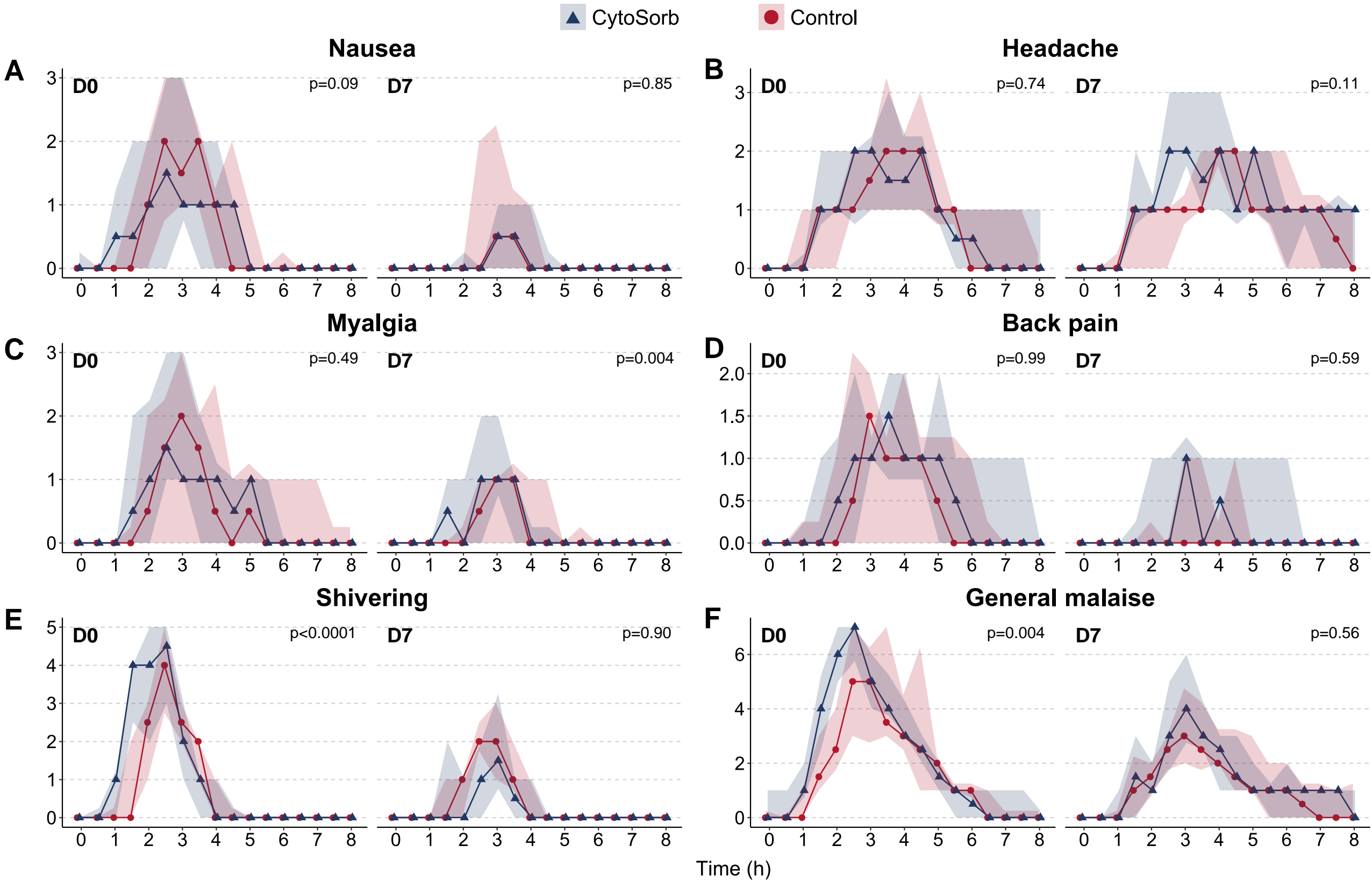

Supplement: Supplementary file 5 — Additional file 5: Fig. S5. Scores of various flu-like symptoms during the first (D0) and second (D7) LPS challenge day. Data are displayed as median (line) and interquartile range (shaded area). P values were computed using two-way repeated measures analysis of variance (time × group interaction term). D0 = day 0, D7 = day 7. [file 13054_2023_4391_MOESM5_ESM.pdf]
