# Supplementary material for: CytoSorb hemoperfusion markedly attenuates circulating cytokine concentrations during systemic inflammation in humans in vivo
Source: Crit Care. 2023 Mar 21;27:117. doi: 10.1186/s13054-023-04391-z (PMC10029173; doi:10.1186/s13054-023-04391-z)
Supplement: Supplementary file 6 — Additional file 6: Fig. S6. Hemocytometry parameters during the first (D0) and second (D7) LPS challenge day. Data are displayed as median (line) and interquartile range (shaded area). P values were computed using two-way repeated measures analysis of variance (time × group interaction term). D0 = day 0, D7 = day 7. [file 13054_2023_4391_MOESM6_ESM.pdf]

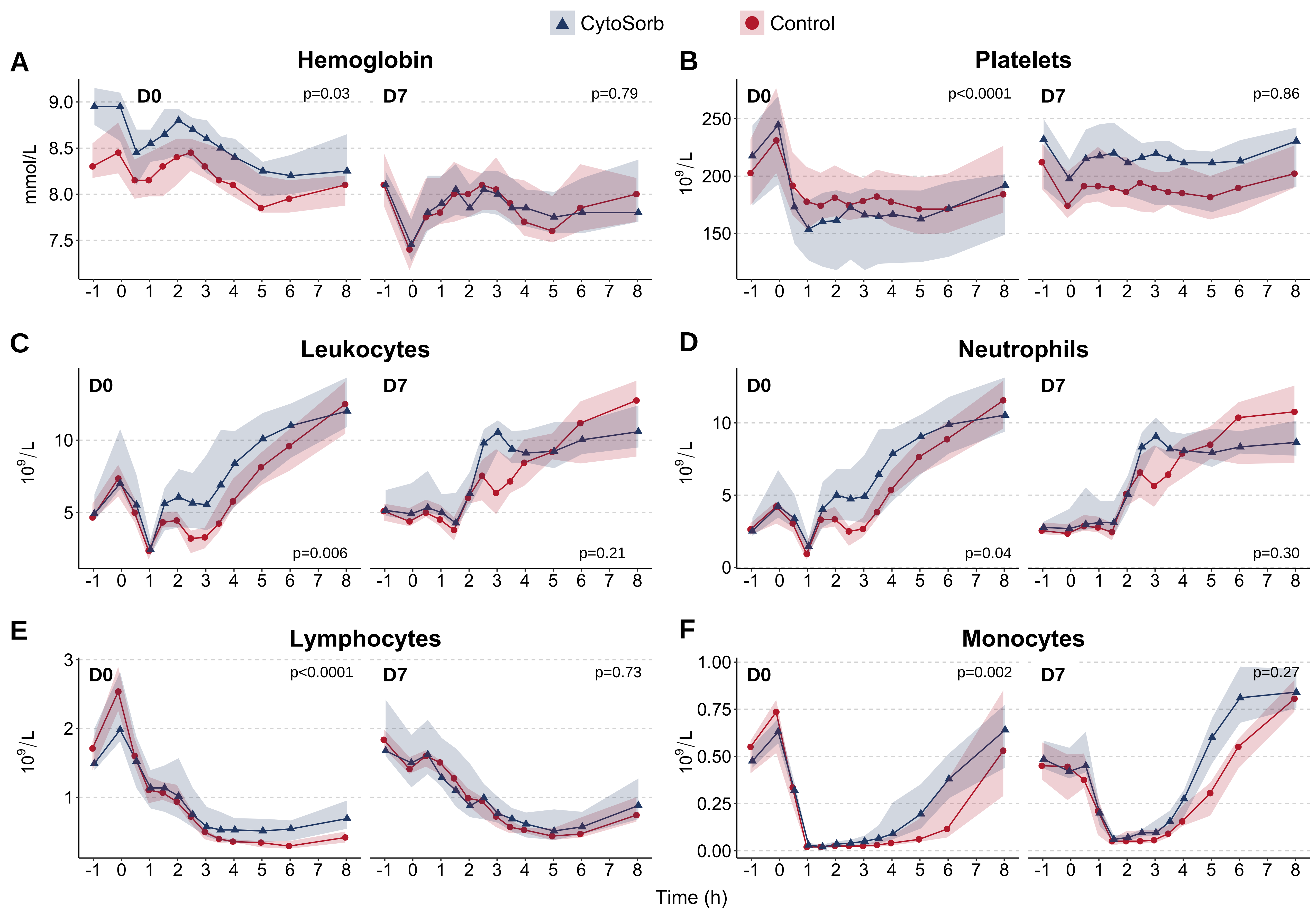

CytoSorb
Control
A
Hemoglobin
mmol/L
D0
p=0.03
D7
p=0.79
B
Platelets
10^9/L
D0
p<0.0001
D7
p=0.86
C
Leukocytes
10^9/L
D0
p=0.006
D7
p=0.21
D
Neutrophils
10^9/L
D0
p=0.04
D7
p=0.30
E
Lymphocytes
10^9/L
D0
p<0.0001
D7
p=0.73
F
Monocytes
10^9/L
D0
p=0.002
D7
p=0.27
Time (h)
